# 

**Published:** 1911-04-15

**Authors:** 


### ANNOUNCEMENTS.

Kentucky State Dental Association. The annual meeting of this Society will be held at Owensboro, May 23 to 25 instead of May 25 to 27, as before announced. All members of the profession are cordially invited.

The Virginia State Dental Association will hold its 42nd annual meeting at the Jefferson, Richmond, Va., June 14, 15, 16, 1911.

There is a very attractive program being arranged and all ethical dentists are invited to attend.

Ohio State Dental Board. The regular spring meeting of the Ohio State Dental Board will be held in Columbus, on June 20 to 23, inclusive. Persons desiring to secure licenses, should address the secretary for an application.

Applications must be returned to the secretary with the fee of twenty-five ($25.00) dollars, not later than June 10th, 1911.

For further information, address the secretary

L. L. Yonker, Bowling Green, Ohio.

National Dental Association Clinics. The Clinic Program for the meeting at Cleveland promises to be an unusually interesting and profitable one. Every effort is being put forth to secure the very best talent as clinicians in the profession.

It is our intention to make this clinic the most interesting and profitable one in the history of the National Association. We have at this early date (March 1st) secured men of national reputations to clinic on the following subjects: Oral Surgery, Prophylaxis, Orthodontia, we hope to arrange a concerted Gold Filling Clinic in cavities in pearl matrices (by 10 operators) under direction of Dr. Southwell of Milwaukee, Progressive Gold and Porcelain Clinics, Gold Inlays and many other demonstrations. Canada and Europe will be represented and we are making great efforts to have every state present their best clinicians. We solicit clinics from any member of State or District Society and a most cordial invitation extended to our Canadian brethern.

Send in your names at once with title of Clinic to any member of the Committee, that we can have the program published in the Journals, June issue.

D. O. M. Le Cron, *Chairman,*
501 Missouri Trust Bldg.

---

A Research Laboratory. A Committee for the Erection of the German "Zahnaerzte-Haus" has been organized in Berlin. The "Zahnaerzte-Haus," an institution founded for the benefit of the dental profession of Germany will contain laboratories for scientific research, a complete dental library, as well as a dental museum, rooms for giving post-graduate courses, and a very complete exhibit of models showing various methods in operative and mechanical dentistry; a collection, which was started some time ago by the state. Besides this, there will be two infirmaries, one for school-children and the other for the poor. Large halls for dental conventions and the meetings of the local societies will be provided for in the building. A bureau will also be incorporated whose duty it will be to collect all statistics bearing upon dentistry, to do the secretarial work of the national dental organizations and benevolent societies, as well, as, to act as a bureau of general information.

It is the earnest wish of the committee to be able to place a library, as complete as possible, at the disposal of the dental profession when the institution is opened. As the collection of such a library must be done at some time, the committee has decided to begin at once, and therefore requests all colleagues who have distinguished themselves by scientific work, to aid in completing this library by donating copies of their works, or reprints. The donors may be assured of the thanks, not only of the committee, but also of all those who have at heart the advancement of dental science. Duplicates, or books to which the owner attaches no special value, would also be gratefully received.

Please address all packages to Zahnarzt Pursche, Ranke Strasse, 30, Berlin, Germany.

Very respectfully yours,

The Committee for the Erection of
German "Zahnaerzte-Haus."

A. Guttman, *Chairman.*
Rob. Richter, D.D.S.
Pursche,
Mamlok,
Helm,
H. W. C. Boedecker, M.D., D.D.S.

---

American Miller Memorial.—Columbus O., March 25, 1911. To the Dental Profession of America: The committee appointed at the December 1909 meeting of the Ohio State Dental Society, for the purpose of raising funds for an American Memorial to perpetuate the memory of the late Dr. Willoughby D. Miller, desires to make the following report:

Honorary Committees have been appointed in the several States, to solicit contributions by which their State may be represented in the fund. It is due to their hearty cooperation that the following report points to a successful issue of this undertaking, to express the appreciation of American dentists for the work of Dr. Miller. The amounts asked for from the several States is prorated according to the membership of the state societies; in some instances these amounts have been partial, while in others they have been slightly over subscribed. The Committee deem it inadvisable, to take steps toward the construction of this memorial until the fund, which will approximate $8,000 is fully subscribed and in the hope this condition will be brought about by Fall.

The following subscriptions are now in the hands of the Treasurer, Dr. Weston A. Price of Cleveland, Ohio. Individual subscriptions while included in the following, will appear in the name of the donor in our final report in the journals. Alabama State Dental Society $25; California State Dental Society $10; San Francisco Dental Society $20; Arkansas State Dental Society $50; Alameda Co., Ca., Dental Society $10; Sacramento Co., Cal., Dental Society $10; Colorado Odontological of Colorado Springs $25; Connecticut State Dental Association $50; Georgia, personal subscriptions and State Dental Society $35; Illinois State Society $150; Illinois Component Societies $216; Illinois personal subscriptions $165; Indiana State Dental Society $50; Eastern Indiana Dental Society $25; Iowa State Dental Society $200; Kansas, personal and State Society $134.50; Kentucky State, Local and personal $105; Montana State Dental Society $15; Nebraska personal and State Society $100; New Hampshire State Dental Society $25; New York Odontological $25; New York Institute of Stomatology $50; South Carolina State Dental Society $25; North Dakota State Dental Society $50; South Dakota State Dental Society $15; Oregon State Dental Society $50; Pennsylvania State Dental Society $200; Lebanon Valley Dental Society Pa., $10; Tennessee State Dental Society $50; Texas State Dental Society $50; Utah, Salt Lake City Aux. Delta Sigma Delta $14; Ohio State Dental Society $1000; Cincinnati Odontographic $100; Columbus Dental Society $100; Northern Ohio Dental Society $200; Virginia State Dental Society $50; West Virginia State Dental Society $25; Washington State Dental Society $50; Wyoming State Dental Society $10; Total $3494.50.

Expenses of our Committee including printing, stationery postage and stenographic work to date amounting to $79.90 has been taken care of by the Ohio State Dental Society.

In addition to the above the State societies of Massachusetts and Missouri have each subscribed $100.00 and Mississippi $50, while several other States have subscribed amounts not definitely reported at this date. Personal subscriptions from Columbus dentists will approximate $500.

Other professions have established memorials to their distinguished dead; dentistry has done it before, and we appeal to your spirit of loyalty and patriotism to aid in honoring this American whose life was one of untiring devotion to the scientific advancement of our profession.

Yours very truly,

EDWARD C. MILLS, *Chairman.*
16 South Third St. Columbus, Ohio.

J. R. CALLAHAN,
25 Garfield Place, Cincinnati, Ohio.

S. D. RUGGLES,
Portsmouth, Ohio.

